# Impact of a Clinical Decision Model for Febrile Children at Risk for Serious Bacterial Infections at the Emergency Department: A Randomized Controlled Trial

**DOI:** 10.1371/journal.pone.0127620

**Published:** 2015-05-29

**Authors:** Evelien de Vos-Kerkhof, Ruud G. Nijman, Yvonne Vergouwe, Suzanne Polinder, Ewout W. Steyerberg, Johan van der Lei, Henriëtte A. Moll, Rianne Oostenbrink

**Affiliations:** 1 Department of general pediatrics, ErasmusMC-Sophia Children’s Hospital, Rotterdam, the Netherlands; 2 Department of Public Health, Center for Medical Decision Making, Erasmus University Medical Centre, Rotterdam, the Netherlands; 3 Department of Public Health, Erasmus University Medical Centre, Rotterdam, the Netherlands; 4 Department of Medical Informatics, Erasmus University Medical Centre, Rotterdam, the Netherlands; Fondazione IRCCS Ca' Granda Ospedale Maggiore Policlinico, Università degli Studi di Milano, ITALY

## Abstract

**Objectives:**

To assess the impact of a clinical decision model for febrile children at risk for serious bacterial infections (SBI) attending the emergency department (ED).

**Methods:**

Randomized controlled trial with 439 febrile children, aged 1 month-16 years, attending the pediatric ED of a Dutch university hospital during 2010-2012. Febrile children were randomly assigned to the intervention (clinical decision model; n=219) or the control group (usual care; n=220). The clinical decision model included clinical symptoms, vital signs, and C-reactive protein and provided high/low-risks for “pneumonia” and “other SBI”. Nurses were guided by the intervention to initiate additional tests for high-risk children. The clinical decision model was evaluated by 1) area-under-the-receiver-operating-characteristic-curve (AUC) to indicate discriminative ability and 2) feasibility, to measure nurses’ compliance to model recommendations. Primary patient outcome was defined as correct SBI diagnoses. Secondary process outcomes were defined as length of stay; diagnostic tests; antibiotic treatment; hospital admission; revisits and medical costs.

**Results:**

The decision model had good discriminative ability for both pneumonia (n=33; AUC 0.83 (95% CI 0.75-0.90)) and other SBI (n=22; AUC 0.81 (95% CI 0.72-0.90)). Compliance to model recommendations was high (86%). No differences in correct SBI determination were observed. Application of the clinical decision model resulted in less full-blood-counts (14% vs. 22%, p-value<0.05) and more urine-dipstick testing (71% vs. 61%, p-value<0.05).

**Conclusions:**

In contrast to our expectations no substantial impact on patient outcome was perceived. The clinical decision model preserved, however, good discriminatory ability to detect SBI, achieved good compliance among nurses and resulted in a more standardized diagnostic approach towards febrile children, with less full blood-counts and more rightfully urine-dipstick testing.

**Trial Registration:**

Nederlands Trial Register NTR2381

## Introduction

Fever is one of the most common symptom among children presenting to the emergency department (ED) [1–3] and accountable for 10–20% of all acute admissions. [4–6] Fever may have various causes, ranging from self-limiting viral infections to serious bacterial infections (SBI) (e.g. septicemia, pneumonia, urinary tract infections). Febrile children at the ED pose a diagnostic challenge, as physicians need to identify that relatively small proportion of SBI with a potential fatal course, in this large group of children with self-limiting diseases.[7–9] To support physicians several guidelines and decision models have been developed focussing on improving diagnosis, limiting diagnostic tests and improved cost-effectiveness. ([4, 7, 10–17] However, the true impact of these diagnostic tools in clinical practice, taking the translation of diagnostic risk predictions to clinical management recommendations and the subsequent compliance of the clinicians with these recommendations into account, is hardly subject of research. [18–20]

In this study we aimed to evaluate the impact of a previously developed clinical decision model for febrile children in the daily practice of the ED. This model includes clinical signs, symptoms and the biomarker C- reactive protein (CRP). [10] Results of this study will bridge the often remaining gap in translating decision models into clinical practice.[19, 21, 22]

## Methods

### Study design and setting

We conducted a randomized controlled trial of a clinical decision model at the emergency department (ED) of the Erasmus MC-Sophia Children’s Hospital in Rotterdam, The Netherlands. The protocol and CONSORT statement are available as supporting information; see S2 and S3 Files. This large inner-city university hospital is visited annually by nearly 9000 children with a mixed ethnic population of which 90% involved basic pediatric care.[2] During the study period there was no national guideline available for handling febrile children at the ED, although most physicians were familiar with the NICE febrile child guideline.[4]

### Study population

We prospectively enrolled all consecutive pediatric patients (≥1 month—<16 years) presenting with fever at our ED from the first of September 2010 until June 30, 2012. Febrile children were eligible if fever had been noted at home in the 24 hours prior to presentation, when body temperature measured at the ED was ≥38.5°C or fever was used as a positive discriminator of the Manchester Triage System (MTS).[23] From the major principle of reducing diagnostic uncertainty by using a diagnostic decision model [5] we excluded well appearing febrile children (no amber/red alarming signs)[4] with a clear focus of uncomplicated rhinitis/otitis and severely ill children (emergent triage category).[23, 24] Children with chronic co-morbidity were excluded because of their increased risk of having serious infections and developing a complicated course.[25] Finally, children who reattended the ED within one week of their first presentation were only included at their initial visit.

### Study intervention

**Fig 1:**
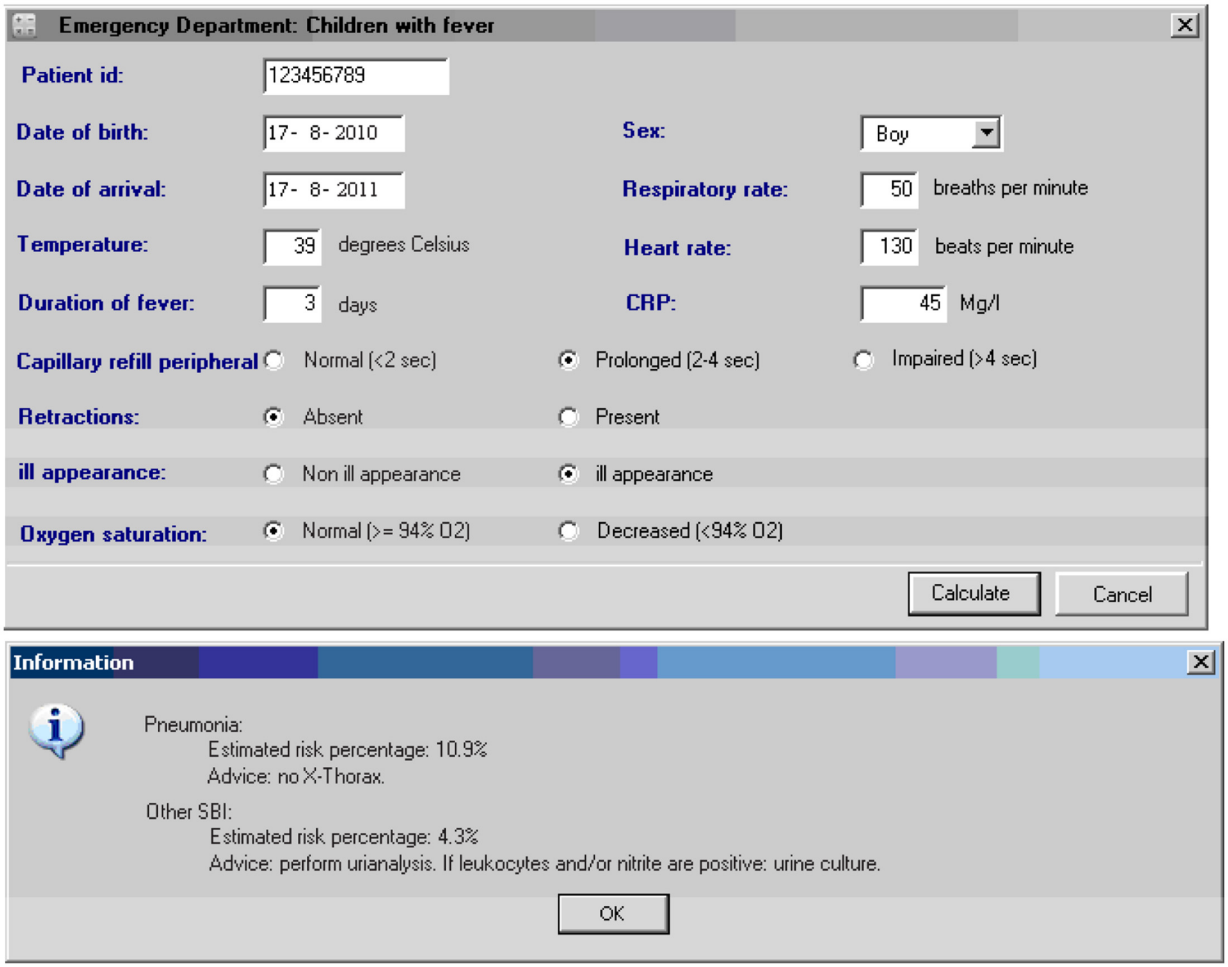
Clinical decision model. Clinical decision model with its predictors (above) and recommendations (below).

The intervention consisted of the implementation of the clinical decision model, based on a polytomous logistic regression model of 2,717 febrile children who presented to the ED and included eleven predictors of pneumonia and other SBI versus no SBI (Table 1)[10]. Instructions on the use of the clinical decision model and the study process included individual briefings, practice cases for emergency staff (nurses/physicians), information by email, and posters mounted at the ED. During the trial period, feedback and periodic teaching sessions were organized. The clinical decision model presented patient-specific risk estimates (percentages) for both pneumonia and other SBI, which were categorized in high- or low risk groups and consequently were accompanied by recommendations for further diagnostic testing (Fig 1). These recommendations and risk estimates were only presented for children allocated to the intervention group; others were designated ‘usual care’ after completion of entering the model predictors (control group). Cut-off points for additional diagnostic testing were based on the diagnostic performance of the original prediction model at different risk thresholds, and were agreed upon by expert panel.[10] For the chosen cut-off points, the expected gain was maximized based on the ratio of false positive/negative errors and expected adverse consequences of a delayed diagnosis SBI. For high-risk children, nurses were instructed to initiate additional testing before physician’s assessment. This recommendation implied chest-radiography in children with estimated risk for pneumonia ≥15% and urine-dipstick and culture if estimated risk for other SBI was ≥30%. Following the local protocol [26] and international guidelines [27] urine-dipstick testing was also recommended in low-risk children without a clear focus for their febrile illness. Nurses and physicians were blinded for these cut-off values and for the contribution of predictors on risk scores. Physicians were able to overrule the recommendations by adding to or refraining from additional diagnostic testing. In the control group the physician first examined the patient and ordered diagnostic procedures according to their own judgment, but with the knowledge of a CRP-value which was required to enable randomization.

**Table 1:** Patient characteristics.

	Intervention	Usual care
	(n = 219)	(n = 220)
*Decision model variables*		
Age (years)^a^	2.0 (1.0–4.2)	1.7 (0.8–3.9)
Sex, male*	140 (63.9)	145 (65.9)
Temperature ^a^ (°C)	38.9 (38.2–39.5)	38.9 (38.0–39.5)
Duration fever^a^ (days)	2.0 (1.0–4.0)	2.0 (1.0–4.0)
Prolonged capillary refill* (>2 sec)	18 (8.2)	26 (11.8)
Chest wall retractions*	10 (4.6)	16 (7.3)
Ill appearance*	44 (20.1)	49 (22.3)
Saturation (<94% O_2_) *	3 (1.4)	4 (1.8)
Respiratory rate ^a^ (/minute)	28.0 (24.0–40.0)	32.0 (24.0–39.0)
Heart rate ^a^ (/minute)	135.0 (120.0–160.0)	140.0 (120.0–156.0)
CRP ^a^ (mg/L)	12.0 (8.0–39.0)	13.0 (7.0–35.8)
*Referral to emergency department*		
Self-referral*	120 (54.8)	115 (52.3)
Primary care*	57 (26.0)	67 (30.5)
Ambulance*	21 (9.6)	17 (7.7)
Other* ^#^	20 (9.1)	20 (9.1)
Missing*	1 (0.5)	1 (0.5)
*MTS urgency*		
Urgent* ^^^	181 (82.6)	193 (87.7)
Non-urgent* ^^^	35 (16.0)	26 (11.8)
Missing*	3 (1.4)	1 (0.5)
*Final diagnoses*		
Serious bacterial infections*	27 (12.3)	28 (12.7)
Sepsis*	1 (0.5)	1 (0.5)
Pneumonia*	19 (8.7)	14 (6.4)
Urinary tract infection*	5 (2.3)	9 (4.1)
Bacterial gastroenteritis*	1 (0.5)	1 (0.5)
Pulmonary tuberculosis) *	1 (0.5)	-
Abscess*	-	2 (0.9)
Scarlatina*	-	1 (0.5)
Self-limiting/ viral diseases*	192 (87.7)	192 (87.3)
Upper respiratory tract infection	109 (49.8)	116 (52.7)
Lower respiratory tract infection	14 (6.4)	15 (6.8)
Gastroenteritis	20 (9.1)	18 (8.2)
Others^^^^	49 (22.4)	43 (19.5)

* Absolute number (percentage).

^a^ Median (25–75 percentiles).

^# ‘^Other’ includes secondary care and after telephone contact.

^**^ ‘**^Urgent’ includes very urgent/ urgent; ‘Non-urgent’ includes standard/ non-urgent.

^^^^ Others includes for example. Influenza, chicken-pox, stomatitis.

### Data collection

All children who attended the ED were routinely triaged with the Manchester Triage System (MTS).[23, 24, 28, 29] After patient triage, model predictors were entered by the nurse for each individual patient. We collected patient characteristics (e.g. gender, age, reason of ED visit), referral profile, duration of the febrile episode, clinical signs/symptoms, observations and measures from physical examination (e.g., vital signs, clinical appearance). Data on all performed laboratory tests (e.g. full blood-count, CRP), additional diagnostic tests (e.g. chest-radiography, urine-dipstick, blood/urine-culture) and treatment/follow-up were registered prospectively in the computer-based hospital information system. The clinical decision model was implemented as a stand-alone device, accessible from each computer at the ED. A central logbook recorded data from all entered participants (Fig 1). Completing all variables of the clinical decision model was mandatory before randomization could be initiated. Once the risk estimate was shown to the nurse, no changes could be made to the entered model predictors.[30] The randomization mechanism, based on even/odd seconds indicated by the digital computer clock, was unknown to nurses and physicians.

Ethics was obtained by the institutional review board (IRB) of the ErasmusMC. According to IRB-review the intervention contained no additional risks as patients were not subjected to additional operations and no rules of conduct were imposed. Written informed consent was required by IRB and it was obtained from all caretakers, or guardians on behalf of the children enrolled (MEC-2008-071).

### Outcome measures

Evaluation of the clinical decision model included diagnostic performance and feasibility of the model. Primary outcome measures were correct diagnoses (SBI) and their related false positive/negative diagnoses by using the clinical decision model in routine practice. Secondly, we measured process outcomes including length of stay (LOS) at the ED and items on diagnostics, treatment and follow-up as defined below.

### Statistical analysis

#### Power analysis

Previous research in the same setting showed that approximately 50% of febrile children at the ED were submitted to diagnostic procedures (blood tests, urine-cultures and chest-radiography).[31] To detect a reduction of unnecessary diagnostic tests from 50% to 35% (false positives) to improve our primary outcome of correct diagnosis, the intervention and control group should include 180 children each (80% power; type-I error of 5%; 2-sided test). This number of patients also allows detecting a difference of 10 minutes patients’ ED length of stay (30 minutes standard deviation (SD)). Adjusted for 10–15% dilution effect by nurses managing both intervention and control patients during the same shift [32] and adjusted for 10% not evaluable cases, the trial planned to include 500 children with fever.

#### Evaluation clinical decision model

We first assessed the performance of the clinical prediction model for our trial population by evaluating discriminative ability according to the area-under-the-receiver-operating-characteristic-curve (AUC) and calibration.[10] Predicted risks of pneumonia and other SBI were compared with the observed proportions of pneumonia and other SBI to assess calibration.[10, 33] Feasibility was measured by compliance of the nurses to the recommendations of the clinical decision model.

SBI were defined according to a reference standard and included abnormal radiographic findings and positive cultures from otherwise sterile body sites (urine, blood, spinal fluid) or excluded by uneventful follow-up by telephone three days after ED discharge. A consensus diagnosis was made if the reference standard was inconclusive (by investigators EK, RGN, RO).[34] [35] All final diagnoses were classified as either pneumonia, other SBI or no SBI. Outcome measures were coded blinded for the allocated randomization arm.

#### Impact analysis

The impact of the decision model was analysed by intention to treat. We evaluated the impact of the clinical decision model by measuring correct diagnoses (SBI) and their related false positive/negative diagnoses,as our primary patient outcome, indicating effects of using the intervention strategy irrespective of overruling or non-compliance by the physician versus the usual care strategy. Primary patient outcomes of the decision model in intervention and usual care were compared using Chi-square analysis. We defined false positives as children without SBI incorrectly exposed to diagnostic tests and false negatives as children with SBI incorrectly refrained from diagnostic tests. We presented sensitivity (children with pneumonia/UTI and performance of chest-radiography/urine-culture), specificity (children without pneumonia/UTI and no performance of chest-radiography/urine-culture) and their related positive/negative likelihood ratios of the complete model. In the control group exposure to testing was based on judgment of the physician only; in the intervention group this was based on the recommendation of the clinical decision model and physicians’ judgment. LOS at the ED was based on the triage starting time and ED departure time as registered in the nursing record; Diagnostic procedures included chest-radiography, urine-dipstick, full blood-count and cultures (blood, urine and others). Treatment/follow-up included antibiotic prescriptions, hospitalization and revisits. Comparisons of process outcomes were tested with Chi-square or Student’s *t* test analysis, *P*-values were two-tailed. All analyses were performed with SPSS PASW statistics software (version 20.0; Chicago, Illinois) and R statistical packages (version 2.14, Vienna, Austria).[36]

## Results

**Fig 2:**
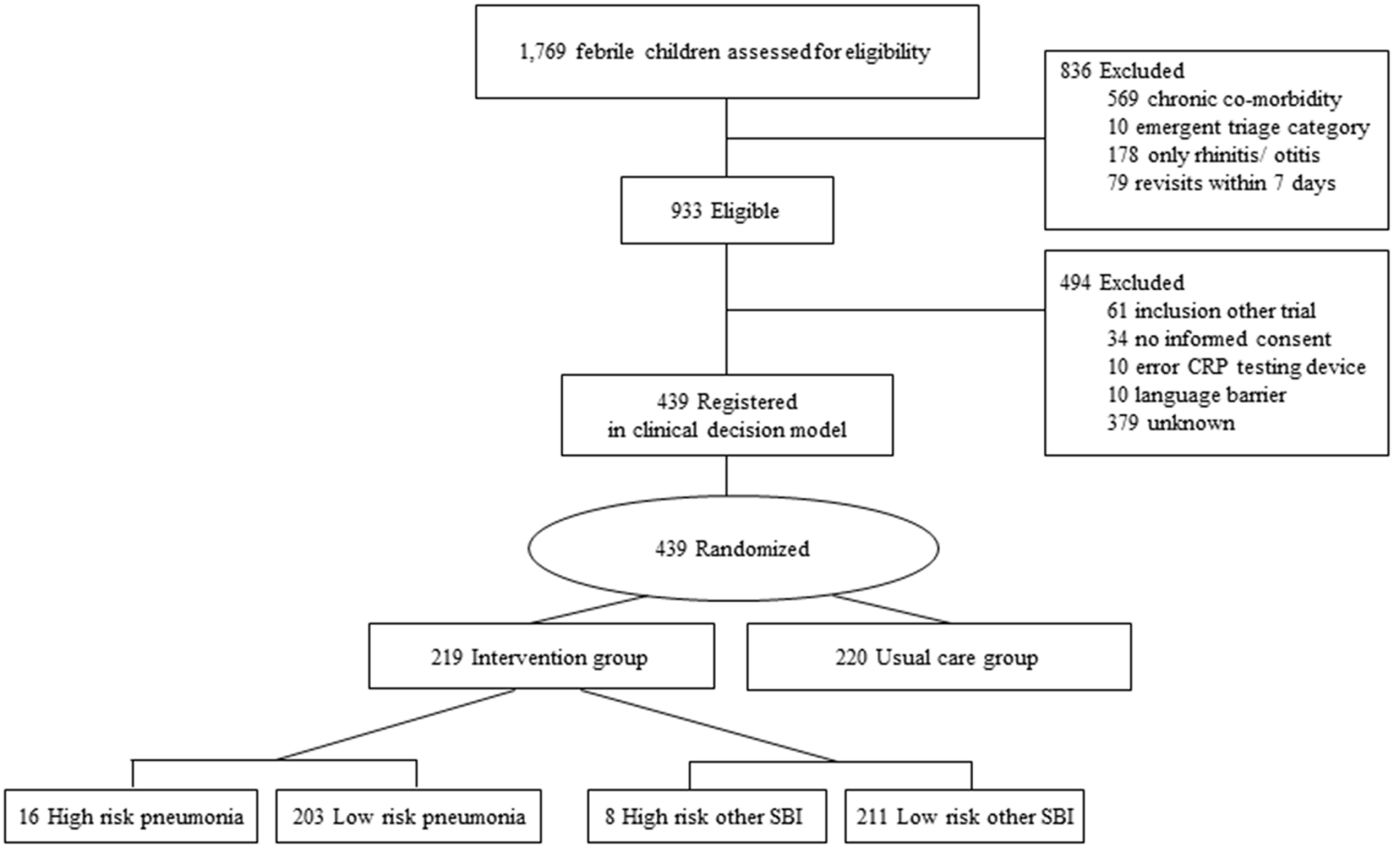
Flowchart patient selection and randomization. Flowchart patient selection and Randomization.

Out of 1,769 eligible febrile children 836 children were excluded because of chronic co-morbidity, emergent triage category, uncomplicated rhinitis/otitis or revisits within 7 days, resulting in 933 eligible children. 439 evaluable children were included for analysis (Fig 2) with a median age of 1.8 years (IQR 0.9–4.1), 57% were boys (n = 249), and the SBI prevalence was 13% (n = 55) including 33 children with pneumonia and 22 children with other SBI (Table 1).

### Evaluation clinical decision model

**Fig 3:**
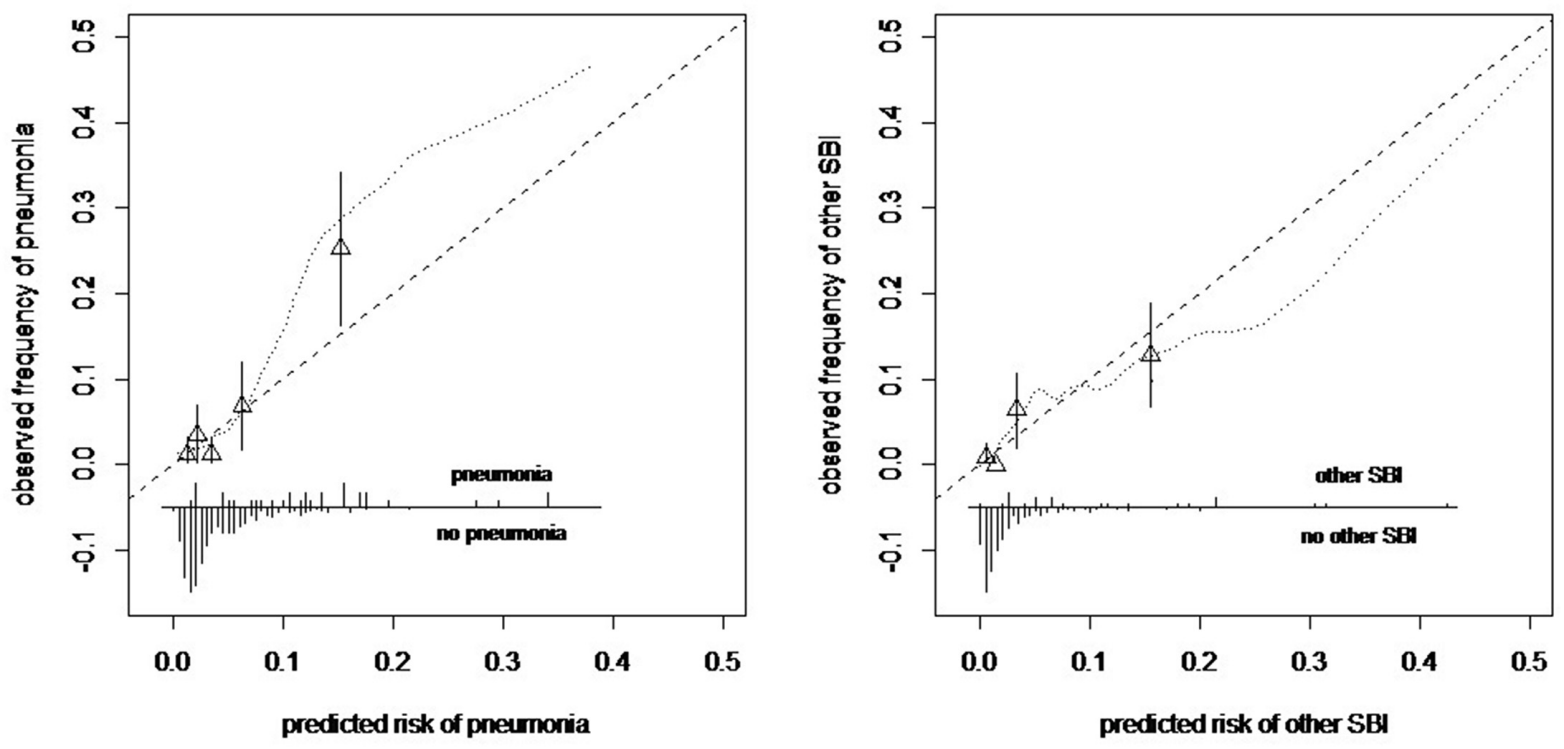
Calibration plot for the risk of pneumonia (left) and other SBI (right). Calibration plot of the predicted risks of pneumonia and other SBI (x-axis) and the observed frequencies of pneumonia and other SBI (with 95% CI, y-axis). The triangles represent the mean (predicted vs. observed) risk estimates of pneumonia and other SBI by quintiles of predicted risk. The dashed diagonal line represents ideal calibration. The distribution of the predicted risks of patients with pneumonia (n = 33) and other SBI (n = 22) and other patients (n = 406 and n = 417) is shown in the bottom of the graph, parallel to the x-axis.

The discriminative ability of the clinical decision model was 0.83 (95% CI 0.75–0.90) for pneumonia and 0.81 (95% CI 0.72–0.90) for other SBI. The clinical prediction model had good calibration in the study population, except for the upper quintiles due to the limited number of cases with pneumonia or other SBI (Fig 3). We also observed limited variability of the predicted risks (SD 0.06 for pneumonia and 0.08 for other SBI) with only 10% of the children (n = 44) being categorized as high risk. Consequently, model recommendations were limited and the majority of children rightly did not undergo diagnostics. 60% (18/30) of the children assigned to high-risk for pneumonia did not have final diagnosis of pneumonia, for other SBI this was 71% (10/14). Five percent (21/409) of children assigned to low-risk for pneumonia had a final diagnosis of pneumonia, for other SBI this was 4% (18/425). All children assigned to high-risk categories with a final diagnosis of SBI received antibiotics (20/20) compared to 57% of high-risk children without final diagnoses of SBI (13/23). Compliance to the recommendations of the clinical decision model in the intervention arm by nurses was high; only three high-risk patients did not receive the recommended diagnostics for either pneumonia (n = 2) or other SBI (n = 1) because of overruling by the physician. In 28 low-risk patients without clear other focus, no urine-dipstick testing was done, leading to a compliance of 86% (188/219).

### Primary patient outcome

In 60% (25/42) of all children allocated to the intervention group chest-radiography was done but no pneumonia was diagnosed (false positives, Table 2). This percentage was not significantly different from the 57% (16/28) false positives of the control group (p = 0.84). In 67% (12/18) of all children in the intervention group urine-culture was collected but no UTI was diagnosed (false positives), but did not differ from the control group (53%, 9/17) (p = 0.41). The clinical decision model performed similarly between both study groups with regard to false negatives (Table 2).

**Table 2:** Patient and process outcome.

	Intervention group	Usual care group
	(n = 219)	(n = 220)
*Patient outcome—pneumonia*		
False positive (no pneumonia/ chest-radiography performed)	25/42 (60%)	16/28 (57%)
Sensitivity to detect pneumonia	0.89 (0.69–0.97)^$1^	0.86 (0.60–0.96)^$2^
Positive likelihood ratio	7.16 (4.81–10.66)	11.0 (6.58–18.81)
False negative (pneumonia/no chest-radiography performed)	2/177 (1%)	2/192 (1%)
Specificity to detect pneumonia	0.88 (0.82–0.91)^$3^	0.92 (0.88–0.95)^$4^
Negative likelihood ratio	0.12 (0.03–0.45)	0.16 (0.04–0.56)
*Patient outcome—other SBI*		
False positive (no UTI/ urine-culture performed)	12/18 (67%)	9/17 (53%)
Sensitivity to detect UTI	1.0 (0.61–1.0)^$5^	0.89 (0.57–0.98)^$6^
Positive likelihood ratio	17.7 (10.2–30.7)	20.8 (10.6–41.1)
False negative (UTI/ no urine-culture performed)	0/201 (0%)	1/203 (0.5%)
Specificity to detect UTI	0.94 (0.90–0.97)^$7^	0.96 (0.92–0.98)^$8^
Negative likelihood ratio	-	0.12 (0.02–0.74)
*Process outcomes*		
1. Patient consultation time		
Time spent at the ED (hrs:min)^a^	1:57 (1:24–2:38)	1:54 (1:21–2:42)
2. Diagnostics		
Chest-radiography	42 (19.2)	28 (12.7)
Urine-dipstick	156* (71.2)	133* (60.5)
Urine-culture	18 (8.2)	17 (7.7)
Full blood-count^#^	31* (14.2)	48* (21.8)
Blood culture	13 (5.9)	20 (9.1)
Other cultures^~^	20 (9.1)	25 (11.4)
Overall^±^	124 (56.6)	138 (62.7)
3. Treatment		
Antibiotics at the ED (iv)	9 (4.1)	14 (6.4)
Antibiotics at discharge (oral)	69 (31.5)	78 (35.5)
SBI_receiving antibiotics_ / SBI_total_	25/27 (92.6)	26/28 (92.9)
no SBI_receiving antibiotics_ / no SBI_total_	44/192 (22.9)	52/192 (27.1)
4. Follow-up		
No	124 (56.6)	137 (62.3)
Hospitalization	26 (11.9)	23 (10.5)
Outpatient clinic	22 (10.0)	27 (12.3)
Telephone call	47 (21.5)	33 (15.0)
5. Safety netting		
Revisit	47 (21.5)	45 (20.5)
Antibiotics after revisit	12 (5.5)	8 (3.6)
Hospitalization	7 (3.2)	5 (2.3)

SBI = serious bacterial infection.

UTI = urinary tract infection.

^a^ Median (25–75 percentiles).

^#^ including hemoglobin, leukocyte, thrombocyte and differential count.

^~^including feces culture, nasal swab, throat culture and cerebrospinal fluid (CSF) culture.

*Chi-square, p-value <0.05.

^±^ Overall diagnostics minus urine-dipstick analysis.

^$1^ In 17 of 19 children with pneumonia chest-radiography was performed.

^$2^ In 12 of 14 children with pneumonia chest-radiography was performed.

^$3^ In 175 of 200 children without pneumonia no chest-radiography was performed.

^$4^ In 190 of 206 children without pneumonia no chest-radiography was performed.

^$5^ In 6 of 6 children with UTI a urine-culture was performed.

^$6^ In 8 of 9 children with UTI a urine-culture was performed.

^$7^ In 201 of 213 children without UTI no urine-culture was performed.

^$8^ In 202 of 211 children without UTI no urine-culture was performed.

### Secondary process outcomes

Median LOS did not differ for children in both the intervention as the control group (1h57min vs. 1h54min, respectively) (Table 2). In the intervention group less full blood-count tests (14% vs. 22%, p-value <0.05) and more urine-dipsticks were done correctly according to current guidelines [26] (71% vs. 61%, p-value <0.05). The number of chest-radiographies and urine-cultures were somewhat higher in the intervention group, although not significant. Overall treatment with antibiotics did not substantially differ between both study groups, (23% in intervention group vs. 27% in control group) (p = 0.30) (Table 2). Finally, no differences in hospitalization or revisits were noted between both study groups.

## Discussion

### Main findings

This impact analysis showed good compliance to the decision model recommendations and its good discriminative ability for detecting SBI was confirmed in febrile children presenting to the ED. Unfortunately, we could not demonstrate improved assessment of correct diagnoses by the intervention. Application of the clinical decision model resulted in a more standardized diagnostic approach towards the febrile child, with significantly less full blood-counts and more rightfully performed urine-dipstick testing in the intervention group.

### Comparison with other studies

This randomized controlled impact trial provides the methodological step after external validation of a developed decision model with good discriminative ability for predicting the presence of pneumonia and other SBI.[10] As an essential step before proven clinical applicability, we performed this impact analysis to test whether or not the decision model actually improved clinical decisions, benefitted patient care or reduced costs.[10, 19–21]

From a literature review it is known that only a minority of new developed clinical prediction models underwent broad validation and no model had undergone impact analysis.[18] The results of our trial were comparable with a recent literature review or individual studies on the effect of these models which showed effects on process outcomes, but only sparse effects on patient outcomes, costs or efficiency.[37, 38] However, this review included only two prediction rules applicable to pediatric emergency care.[31, 39]

### Clinical and research implications

Although our study reports good compliance to and high accuracy of the clinical decision model, positive impact on routine care was lacking. A number of reasons have been described that explain why an accurate clinical prediction model may not result in improved patient care and processes.[40–42] One important reason is that physicians’ intuitive estimation of probabilities may be as good as, if not better than, the prediction model. This is reflected by high positive and negative likelihood ratios [8] in the detection of pneumonia/ UTI for both the intervention as the usual care group (Table 2). Clinicians approach in febrile child management is already on the safe side, as reflected by the absence of worse primary outcomes which was part of our primary outcome. As in practice one would never accept increased false negative diagnoses, it can be considered that trials as ours can only have effects on secondary (process) outcomes like diagnostic tests and treatment. Next, it can be argued that studying improved patient outcomes in febrile children is difficult anyway, with decreasing prevalence of the most severe diagnoses as sepsis and meningitis.[43] Furthermore, this implementation trial was conducted at a highly specialized and experienced university pediatric ED, with residents and experienced supervising pediatricians. We expect that future use of the clinical decision model in a more general setting with less specialized medical staff may lead to improved process outcomes.

One major influence explaining the lack of impact on LOS was probably the availability of CRP bedside-testing in both study groups. Previous research showed that CRP bedside-guided decision making reduced the LOS at the pediatric ED by 19% (median LOS 148 minutes).[25] The rationale for testing CRP in all children was already discussed before in the original paper.[10] For this trial we wanted to test the effect of the decision model as a whole, rather than testing the individual effect of CRP. Finally, we completed our impact trial with a cost-minimisation study (S1 File). Costs of our process outcomes (e.g. blood counts, urine analysis) were low and no difference were found in e.g. hospitalization rate or costs of adverse events. As a consequence, neither this impact analysis, nor a sensitivity analysis could demonstrate substantial cost savings of using the clinical decision model compared with usual care management.

Compliance to the recommendations of the clinical decision model was good. However, we did not include 379 children for unknown reasons, which may suggest some feasibility problems of the decision model itself, but also reflects the difficulties of performing a randomized controlled trial (with the need of informed consent) in emergency care. As we did not observe significant differences on median age (1.9 (IQR 2.0–3.0) vs. 1.8 (IQR 2.0–3.0)), gender (55.7% males vs. 56.7% males) and prevalence of SBI (14.2% vs. 12.5%), when we compared non-included with included patients, we feel that this most likely did not affect generalizability of our results. Another measure of feasibility could be whether the model variables were completed directly after the patient’s triage. This was not the case for all patients, as some were seen by a physician directly during the triage process of the nurse.[5, 23] In more densely crowded ED settings that require triage systems to truly prioritize physicians’ tasks, higher efficiency due to the implementation of the decision model can be expected.

### Strengths and limitations

The main strength of this study is its randomized design to evaluate the last step required for translating prediction research into clinical practice. Second, as we modelled children’s age in a linear piecewise manner,[10] we accounted for the differentiated risks and the uniqueness of signs and symptoms of children in the broad included age group. A subgroup analysis on children aged ≤36 months (n = 287; 65%), who are considered the primary focus in many diagnostic fever studies [44], had similar conclusions. Next, we created optimal conditions for the implementation before we started our impact trial.[45] First, we ensured the compliance of the nurses to the computerized patient triage system at our ED was already high (90% compliance and 97% adherence to the MTS advice).[46] Second, there was an electronic patient record for nurse’s evaluation and, the clinical decision model was implemented in the routine workflow at the pediatric ED. Thirdly, involvement of the nurses during the process of tailoring the format of decision models to the local circumstances resulted in willingness to adopt the system in routine care.[47] Finally, we incorporated a structured follow-up by telephone for ruling-out the possibility of missed and clinically relevant SBI diagnoses in children not fulfilling reference standard criteria. This helps us to reduce verification bias, and is recommended as a valid proxy for missing reference tests [48].

Our study has some limitations. First, reference tests were ordered based on the recommendations of the clinical decision model or at the physician’s discretion. However, to reduce verification bias we used a standardized follow-up period to ensure no clinically relevant SBI were missed in children who did not have chest-radiography or specimens bacterial culture.[48, 49] In addition, as antibiotic prescription is relatively low in our study (around 30%), limiting the number of children with true SBI who had no reference standards done by pre-emptive antibiotics use, we can assume that associated false negative diagnoses were low. Second, some interobserver variability might have influenced the validity of the clinical decision model.[50] As the discriminative ability of the model was good in this study, as in previous settings [10] and the model included mainly vital (relative objective) signs, this limitation has probably not influenced our results negatively. Third, although the clinical decision model was integrated in the nurses’ regular work-flow as stand-alone software, optimal implementation would have been a fully integrated system in the computer-based hospital information system including automatic pop-up screens and reminders. Fourth, from the perspective to focus on febrile children with the largest diagnostic uncertainty, selection criteria differed slightly from the original population in which the model was derived. As the validity was preserved in this new (randomized) population, selection of slightly different patients won’t have affected results, but may have some consequences for generalization of results to other settings.

Finally, our clinical decision model advised on performing chest-radiographies which is in contrast with recent advice of the British Thoracic Society [51] on limited use of chest-radiographies in suspected childhood pneumonia. As the model fairly classifies high/low risk of SBI, we expect it can validly guide decisions on appropriate treatment as well. This might be supported by the lower unnecessary antibiotic prescriptions in the intervention group. Therefore, future recommendations of the clinical decision model may preferably focus on impact on therapeutic management like antibiotic prescription.

## Conclusion

This impact analysis translates a validated clinical prediction model for febrile children into clinical practice. Although we observed well implementation, no substantial impact on patient outcome was perceived. Further evaluation may focus on impact in other settings with greater variability in experience in the assessment of the febrile child, in patient numbers, and in prevalence of outcome measures, or may include guidance on therapeutic management.

## Supporting Information

S1 File Economic evaluation. (DOC)

S2 File Trial register protocol. (DOC)

S3 File CONSORT statement. (DOC)
