# Supplementary material for: Impact of a Clinical Decision Model for Febrile Children at Risk for Serious Bacterial Infections at the Emergency Department: A Randomized Controlled Trial
Source: PLoS One. 2015 May 29;10(5):e0127620. doi: 10.1371/journal.pone.0127620 (PMC4449197; doi:10.1371/journal.pone.0127620)
Supplement: S1 File — (DOC) [file pone.0127620.s001.doc]

**Supplemental file 1: economic evaluation**

**Methods**

Economic evaluation was performed from the hospital perspective. Medical costs were calculated by multiplying the volumes of health care use with the corresponding unit prices.

In particular, we were interested in the impact on process outcomes of diagnostic tests and antibiotic prescription as these procedures were supposedly susceptible to implementation of the clinical decision model. We used real unit prices when available; otherwise charges were used as a proxy for real costs. In-hospital medical costs included costs per patient hour for each caregiver, initiated diagnostics and treatment, length of stay at the ED, hospitalization and revisits. Volumes of diagnostics and treatment were measured according the computer-based hospital information system. Effects of the clinical decision model were differences in the number of false positive and false negative rates. When the clinical decision model resulted in improved patient outcomes with reduced medical costs, complete cost-effectiveness analyses would not be necessary. If the clinical decision model resulted in similar patient outcomes a cost-minimization study was required. Finally, if patient outcomes were worse but with reduced costs, a complete cost-effectiveness analysis was necessary. A sensitivity analysis was performed for differences in the prevalence of SBI where our actual prevalence was increased and decreased with 20%. In addition, we tested the assumption that chest-radiography was no longer routinely applied in children suspected for a lower respiratory tract infection, as recently recommended in guidelines of the British Thoracic Society.^43^ Finally, cost differences of 20% variation in costs of diagnostic tests and treatment/ follow-up were calculated. Differences in future health effects were not included and discounting was not applied because of the limited time horizon.

**Results**

A cost-minimization study was performed because no differences in patient outcomes were observed. Development and implementation of the clinical decision model were estimated to cost €7000 (**table 1**), which was 6% of the total costs in the intervention group. The costs of an ED visit for a febrile child was on average €475 in the usual care group and €525 in the intervention group, excluding development and implementation costs of the clinical decision model. Costs of chest-radiographies amounted for 3 to 5% of the total average costs and for 9 to14% of the total diagnostic costs for febrile children in both study groups. **Table 2** presents the results of the sensitivity analysis compared to the base-case analysis (**table 1**) with an average healthcare cost-difference per febrile child of €50. All assumptions tested in the sensitivity analysis, excluded development and implementation costs of the clinical decision model and resulted in no or only small relative cost-differences. In detail a 20% increase or decrease in the prevalence of SBI resulted in a difference of the total average costs per patient of €41 to €60, respectively. Under the assumption that no more chest-radiographies were performed for the diagnosis of pneumonia we could save €43 per febrile patient. If all costs for diagnostics were over- or underestimated by 20%, the difference in total average costs per patient as the relative cost difference did not change (€52 - €50). Finally, 20% over- or underestimation of the costs of treatment and follow-up resulted in a difference in total average costs per patient of €60 to €42, respectively (**table 2**).

**Table 1 Average health care costs (Euros) per patients for intervention and usual care**

| **Cost category** | **Intervention (n=219)**  **Cost price Volume Costs** | | | **Usual care (n=220)**  **Volume Costs** | |
| --- | --- | --- | --- | --- | --- |
|  |  |  |  |  |  |
| *Clinical decision model* | | | | | |
| Development (hours) | 36 | 144 | 5,184 | - | - |
|  |  |  |  |  |  |
| *Implementation* |  |  |  |  |  |
| Researcher (hours) | 54 | 4 | 216 | *-* | *-* |
| Nurses^*^ (number*hours) | 40 | 20*2 | 1,600 | *-* | *-* |
|  |  |  |  |  |  |
| *CRP testing device* |  |  |  |  |  |
| Depreciation (years) | 138 | 2 | 276 | - | - |
| Maintenance (years) | 246 | 2 | 492 |  |  |
|  |  |  |  |  |  |
| *ED visit* |  |  |  |  |  |
| Physician^ (hrs*number) | 0.25*68 | 219 | 3,723 | 220 | 3,740 |
| Nurses (hours*number) | 0.33*40 | 219 | 2,891 | 220 | 2,904 |
| Hospital costs | 114 | 219 | 24,966 | 220 | 25,080 |
|  |  |  |  |  |  |
| *Diagnostics* |  |  |  |  |  |
| Urine dipstick | 3 | 156 | 468 | 133 | 399 |
| CRP rapid testing | 5 | 219 | 1095 | 220 | 1100 |
| Hematology^#^ | 6 | 31 | 186 | 48 | 288 |
| Blood culture | 24 | 10 | 240 | 16 | 384 |
| Urine culture | 3 | 18 | 54 | 17 | 51 |
| Other cultures~ | 19 | 20 | 380 | 25 | 475 |
| X-thorax | 129 | 42 | 5,418 | 28 | 3612 |
| Bladdercatheterization |  |  |  |  |  |
| Material | 58 | 7 | 406 | 8 | 464 |
| Time physician | 60 | 0.25*7 | 105 | 0.25*8 | 120 |
|  |  |  |  |  |  |
| *Medication* |  |  |  |  |  |
| Antibiotics at ED (iv) | 7 | 9 | 56 | 14 | 98 |
| Antibiotics at discharge (oral) | 2 | 69 | 138 | 78 | 156 |
|  |  |  |  |  |  |
| *Follow-up / hospital stay* | | | | | |
| Length of stay in hospital (days*number) | 575 | 3.5*26 | 52,325 | 3.5*23 | 46,288 |
| Outpatient clinic | 129 | 22 | 2,838 | 27 | 3,483 |
| Telephonic follow-up | 20 | 47 | 940 | 33 | 660 |
|  |  |  |  |  |  |
| *Costs of diagnoses/ adverse events* | | | | | |
| Revisit ED | 144 | 47 | 6,768 | 45 | 6,480 |
| Readmission length of stay (days*number) | 575 | 3*7 | 12,075 | 3*5 | 8,625 |
|  |  |  |  |  |  |
| *Total costs*  *Total costs per patient* |  |  | *122.840*  *561* |  | *104.407*  *475* |
| *Costs without development and implementation of clinical decision model* | | | | | |
| *Total costs*  *Total costs per patient* |  |  | *115,072*  *525* |  | *104.407*  *475* |

^*^ including nurses training in CRP bedside testing/ device

^ 80% resident (60) en 20% physician (100)

^#^ including hemoglobin, leukocytes count, platelets count and differential count

~ including feces culture, nasal swab, throat culture and cerebrospinal fluid (CSF) culture

**Table 2 Sensitivity analyses: total average healthcare costs per febrile child**

|  | Intervention  (n=219) | Usual care  (n=220) | Cost-difference | Relative cost-difference |
| --- | --- | --- | --- | --- |
|  |  |  |  |  |
| Base-case analysis | 525 | 475 | 50 | 10% |
|  |  |  |  |  |
| SBI prevalence +20% | 538 | 497 | 41 | 8% |
| SBI prevalence -20% | 513 | 453 | 60 | 12% |
|  |  |  |  |  |
| No chest-radiographies | 501 | 458 | 43 | 9% |
|  |  |  |  |  |
| Costs of diagnostics +20% | 533 | 481 | 52 | 10% |
| Costs of diagnostics -20% | 518 | 468 | 50 | 10% |
|  |  |  |  |  |
| Costs of therapeutics/ follow-up +20% | 594 | 534 | 60 | 10% |
| Costs of therapeutics/ follow-up -20% | 457 | 415 | 42 | 9% |
|  |  |  |  |  |
|  |  |  |  |  |

SBI: serious bacterial infections
