# Supplementary material for: Impact of a Clinical Decision Model for Febrile Children at Risk for Serious Bacterial Infections at the Emergency Department: A Randomized Controlled Trial
Source: PLoS One. 2015 May 29;10(5):e0127620. doi: 10.1371/journal.pone.0127620 (PMC4449197; doi:10.1371/journal.pone.0127620)
Supplement: S2 File — (DOC) [file pone.0127620.s002.doc]

**TRIAL REGISTER**

**NTR 2381**

**PUBLIC TITLE**

Clinical decision support in children with fever.

Impact of clinical decision support on paediatric emergency care.

**SCIENTIFIC TITLE**

Clinical decision support in children with fever.

Impact of clinical decision support on paediatric emergency care.

**HYPOTHESIS**

Evidence based medicine is the standard for current clinical practice. Guidelines and decision rules are developed to support medical decision making. Application of decision rules are supposed to improve efficiency and quality of care by early recommendations on diagnostic tests or treatment. In the process of translating prediction rules into practice impact analysis is required to show whether or not the decision rule actually improves clinical decisions and will benefit patient care or reduces costs^1^. Children with fever are responsible for about 50% of the paediatric emergency admissions^2^. These children pose a diagnostic challenge at the paediatric emergency department (ED), since fever may have various causes, ranging from serious bacterial infections to self-limiting viral infections^3^. We now introduce a clinical decision support system (CDSS) focusing on this acute paediatric problem with challenging diagnostic dilemmas. We will evaluate a diagnostic decision rule to discriminate children with fever because of a serious bacterial infection from those with self-limiting infections.

Study results are expected to determine the optimal diagnostic strategy of children with fever at risk for serious bacterial infections (SBI) at the paediatric emergency department.

We hypothesize an increase in the number of correct diagnosis (feverish children with serious bacterial infections) and a decrease of false positive errors (children without the target diagnosis incorrectly exposed to diagnostic tests). We hypothesize that the patient’s consultation time will be shorter and less diagnostics and therapeutics are performed. Last we hypothesize children will be less hospitalized, follow-up consultations and costs of these process outcomes will be reduced.

^1^ Reilly et al. Ann. Intern Med. 2006;144:201-9

^2^ Bouwhuis et al. Ned Tijdschr Geneeskd. 2001;145:1847-51.

^3^ Bleeker et al.Acta Paediatr. 2007; 96:100-4

**HEALTH CONDITION/PROBLEM STUDIED**

In the Netherlands approximately 500.000 children attend hospital emergency departments each year. Children with fever are responsible for about 50% of the paediatric emergency admissions^1^. These children pose a diagnostic challenge at the paediatric emergency department (ED), since fever may have various causes, ranging from serious bacterial infections (e.g. meningitis, septicaemia, pneumonia, urinary tract infection) to self-limiting viral infections^2^.

Currently, these patients are observed and triaged by the nurse, then evaluated by the physician, where after diagnostic tests, treatment, follow-up and/or hospital admission are applied. In routine care, about half of the children with fever admitted to the emergency department undergo diagnostic tests, but only 12-15% suffer from serious bacterial infections^3^. Early discrimination of children with fever at high risk for serious bacterial infections will reduce patient consultation time, diagnostic tests and costs in low risk patients, without missing cases with serious bacterial infections.

Given the widespread nature of the problem, and the availability of the technology and scientific method, this study will answer the relevant questions on patient outcome and cost-effectiveness of a clinical decision support system and potentially leading to improved patient care and efficiency.

^1^ Bouwhuis et al. Ned Tijdschr Geneeskd. 2001;145:1847-51.

^2^ Bleeker et al.Acta Paediatr. 2007; 96:100-4

^3^ Roukema et al. JAMIA 2008; 15:107-13

**INCLUSION CRITERIA**

Children aged 1 month - 16 years, visiting the paediatric emergency department of the ErasmusMC-Sophia with fever.

**EXCLUSION CRITERIA**

Fever with a clear focus of only rhinitis or otitis. Children with emergent triage category according to the Manchester Triage System (MTS). Children with chronic morbidity, like: ccystic fibrosisystic fibrosis; bbroncho-pulmonaire dysplasieroncho-pulmonary dysplasia; nnefrotisch syndroom en nierinsufficiëntieephrotic syndrome and renal insufficiency; ppsychomotore retardatie met minimaal rolstoelafhankelijkheid (Gross Motor Function Classification Scale IV en V)sychomotor retardation (Gross Motor Function Classification Scale IV and V); sshort bowel syndroomhort bowel syndrome; maligniteit, waarvoor thans in behandelschemamalignancy currently in treatment regimen; immuundeficiëntie, neutropenieimmune deficiency, neutropenia;gebruik immuunsuppressiva (prednison, enbrel) using immunosuppression medication; hemodynamische instabiele hartafwijkinghaemodynamic unstable heart disease.

**INTERVENTIONS**

At the moment of triage, variables included in the decision rule for children with fever are documented in the electronic patient record by the nurse. Based on these data, the decision rule will classify children into high or low risk of serious bacterial infections (modeled separated for ‘Pneumonia’ en ‘SBI others’). In the low risk group, the rule will recommend no further diagnostic testing, in the high risk group additional testing (X-ray/ urine culture) is recommended. Children with fever are randomly assigned to either the recommendations of the decision rule (intervention group) or the usual care (control group) at the moment of patient triage. Recommendations will only appear to the nurse in the intervention group. Actions are initiated by the nurse directly. Finally, in addition to the recommended tests by the rule, patients are evaluated by the physician similarly to the control group in order to assess underlying correct diagnosis. In the control group, any diagnostic test will be initiated after evaluation by the physician.

1. Arm 1: intervention arm (= 'diagnostic decision rule')

The 4 options of the ‘model’:

Risk of Pneumonia low: no X-ray is performed by the nursing staff

Risk of Pneumonia high: nursing staff arranges a X-ray

Risk of ‘SBI other’ low: nursing staff arranges a urine dipstick

Risk of ‘SBI other’ high: urine culture through catherization/ midstream urine

1. Arm 2: usual care (controls)

**PRIMARY OUTCOME**

Patient outcome:

1. Correct diagnosis (feverish children with serious bacterial infections)
2. False positive errors (children without the target diagnosis incorrectly exposed to diagnostic tests)
3. False negative errors (children with the target diagnosis incorrectly refrained from diagnostic tests).

**SECUNDARY OUTCOME**

Evaluation of the clinical decision model including diagnostic performance and feasibility (compliance to the recommendations of the model).

Process outcome: patient's consultation time spent at the emergency department; the number of diagnostic procedures (chest radiography, blood, urine and liquor tests and cultures); treatment procedures (antibiotic prescriptions), number of hospitalizations; follow up consultations within 1 week.

Costs: costs of diagnostic tests at the emergency department visit, initiated treatment, hospitalization, costs of missed diagnosis and their adverse events, costs of emergency care nurses and physicians.

**TIMEPOINTS**

***Timepoint 0***: All children are routinely evaluated by ED nurses during there visit to the emergency department. Patient characteristics, symptoms, observations and measures from physical examinations are registered by the nurse in a structured data entry application (SDE), as well as working diagnosis, final diagnosis and follow-up appointments.

***Primary outcome:*** The correct presence of serious bacterial infections, i.e. septicaemia, meningitis, pneumonia, urinary tract infection, osteomyelitis, are defined by a positive bacterial or viral culture or consensus diagnosis. When a reference test is not performed, a follow-up contact (visit or telephone call) within a week defines the absence of a serious bacterial infection. False positive errors are children with fever not suffering from serious bacterial infections, who incorrectly undergo additional diagnostic tests and cultures. False negative errors refer to children with fever suffering from serious bacterial infections, who incorrectly are refrained from any diagnostic testing.

***Secundary outcome***

Patient's consultation time, the number of diagnostic tests and treatment, hospitalisation and follow-up consultations are measured using the SDE application.

***Costs*** are estimated on resource use and unit costs and include emergency care visit, health practitioner activities, diagnostic procedures, medical treatment, inpatient days in hospital and costs of adverse event of missed diagnosis (unscheduled readmissions, prolonged hospitalization, increased diagnostic test).

**BRIEF SUMMARY**

OBJECTIVE

To assess the impact of a diagnostic decision rule for children with fever at risk for serious bacterial infections on patient outcome and costs.

STUDY DESIGN

We will perform a randomized controlled trial on the cost-effectiveness of a diagnostic decision rule for children with fever presenting at the emergency department. Randomization will be at individual patient level.

STUDY POPULATION

Children aged 1 month to 16 years visiting the emergency department of the ErasmusMC-Sophia Children's Hospital with fever. Children with chronic morbidity are excluded.

INTERVENTION

We will evaluate a diagnostic decision rule to discriminate children with fever because of a serious bacterial infection from those with self-limiting infections.

OUTCOME MEASURES

Evaluation of the model: diagnostic performance and feasibility.

Patient outcome: correct diagnosis, false positive errors and false negative errors.

Process outcomes: Patient's consultation time, Number of diagnostic tests and treatment, number of hospitalizations; follow up consultations within 1 week.

Costs of process outcomes.

POWER ANALYSIS

To detect a reduction of unnecessary diagnostic tests (false positive rates) from 50% to 35% with an 80% power and a type I error of 5%, the usual care and intervention group should include 180 children each. This number of patients also allows detecting a difference of 10 minutes patients’ consultation time at the ED (30 minutes standard deviation). Adjusted for 10-15% dilution effect by nurses managing both intervention and control patients during the same shift and adjusted for 10% not evaluable cases, the trial planned to include 500 children with fever.

IMPACT ANALYSIS

Patient outcome of the decision rule is assessed by comparing the number of false positives and false negative errors in patients assigned to the decision rule with the patients assigned to usual care. The actual patient outcome is compared with the potential patient outcome based on the rule's recommendations regardless of implementation. Preserved validity of the original prediction rule is evaluated by comparing the rule's discriminative value in the new population to this value in the original population.

ECONOMIC EVALUATION

Cost-effectiveness is assessed by calculating the incremental cost-effectiveness ratio. Analysis will be performed from the hospital perspective. Effects are differences in the number of false positive and false negative errors, where false positive errors will be weighted as clinically less important than false negative errors. Costs will be estimated on resource use and unit costs.

TIME SCHEDULE

Dec 2009 - June 2010: integrating the decision rule with the electronic patient record and triage system. Introduction of the decision rule to the paediatric emergency nursing staff. Training of the paediatric emergency nursing staff to apply the decision rule.

July 2010 - July 2012: prospective randomized application of the decision rule with randomization at individual patient level.

August 2012 - Okt 2012: impact analysis and cost-effectiveness analysis, publication of the results.
